# Immediate IUD insertion after second trimester abortion: implications for service delivery

**DOI:** 10.1186/s12913-021-07306-2

**Published:** 2021-12-04

**Authors:** O. Somefun, D. Constant, M. Endler

**Affiliations:** 1 Women’s Health Research Unit, University of Cape Town, Anzio Road, 7925 Observatory, Cape Town, South Africa; 2 Department of Women and Children’s Health, Karolinska Institutet, Stockholm, Sweden

**Keywords:** Second trimester medical abortion, Copper IUD, Immediate insertion, Implications for service delivery, South Africa

## Abstract

**Background:**

The availability of modern contraception including long-acting reversible contraceptives (LARC), is a fundamental component of postabortion care. Findings from a recent randomized controlled trial (RCT) in South Africa comparing immediate to delayed insertion of the copper intrauterine device (IUD) after medical abortion (MA) at 17-20 gestational weeks showed that immediate insertion resulted in higher IUD use at 6 weeks postabortion, but that expulsion rates were significantly higher than for delayed insertion. This study aims to explore barriers, facilitators, and context-specific factors relevant to the implementation of immediate IUD provision after second trimester medical abortion.

**Methods:**

We performed a qualitative study alongside the RCT in which we conducted in-depth interviews with 14 staff providing healthcare to study participants and 24 study participants. Research questions explored barriers and facilitators to implementation of immediate IUD insertion, contraceptive decision-making, and the impact of context and supplementary trial activities on service provision. Interviews were recorded and transcribed, with translation into English if needed. We performed a triangulated thematic analysis at the level of the transcribed interview text.

**Results:**

Contraceptive counselling at the abortion facility by a study nurse improved knowledge, corrected misconceptions, and increased demand for the IUD postabortion. Women expressed a clear preference for immediate insertion. Convenience, protection from pregnancy and privacy issues were paramount and women expressed preference for engagement with staff who knew their abortion history, and with whom they had an established connection. Doctors and nurses were generally in favour of immediate insertion and said it could be incorporated into standard care if women wanted this. This contrasted with the need for interventions by the research team to reinforce adherence by staff to provide contraception as allocated during the trial.

**Conclusions:**

Women and staff favour immediate IUD insertion after second trimester medical abortion, but service delivery may require structures that ensure timely insertion postabortion, continuity of care, communication that mitigates loss to follow-up and training of staff to ensure competence.

## Background

Comprehensive abortion care, including postabortion contraception, reduces recurring unintended pregnancies, increases birth spacing and improves maternal health [1]. Among South African women having a pregnancy over a five-year period, 66% reported the pregnancy was unintended [2]. While long-acting reversible contraception methods (LARC) such as the IUD are most effective for planning and spacing pregnancies [3], use of the IUD in South Africa is low, at 2-3% [2], despite free provision in the public sector.

Barriers to effective contraceptive uptake and use persist in South Africa and include poor provider attitudes, lack of training in LARC insertion, long waiting hours, little or no contraceptive counselling, and high rates of discontinuation due to both personal and structural factors [4]. Healthcare centres mandated to provide postpartum and postabortion contraception are routinely overburdened resulting in short staff-to-patient interactions and quick-fix contraceptive provision.

Specific to the IUD, barriers to use involve both provider and client attitudes. Misconceptions and negative perceptions of the IUD are common among potential users in South Africa [5, 6]. Providers of contraception have inadequate and sometimes incorrect knowledge and express discomfort with insertion, favouring other methods that take less time to administer [5]. Recent training programs to improve access to the IUD postpartum have shown promise in Southern Africa [6, 7], however generally, uptake remains low [2].

Between 2018 and 2019, we conducted a randomized controlled trial (RCT) in South Africa comparing safety, effectiveness, and acceptability of immediate to delayed insertion of the Copper IUD after medical abortion at 17-20 gestational weeks. We found significantly higher rates of IUD use at 6 weeks, 3 and 6 months after immediate insertion, in large part because women failed to attend their local healthcare facility for delayed insertion. However, as was expected, expulsion rates after immediate insertion were also higher [8]. The RCT was registered at clinicaltrials.gov/ (ID NCT03505047), and on the Pan African Trials Registry (www.pactr.org), ID PACTR201804003324963.

During the RCT we saw unanticipated decision-making by clinical staff providing the postabortion contraception, and by women with respect to method continuation or switching at the 6-week and 3-month postabortion follow-up study visits. These findings warranted further research to address the feasibility of implementing IUD services in this and other similar clinical settings. This qualitative study aims to inform policy makers, program managers and healthcare providers of barriers, facilitators, and context-specific factors relevant to the implementation of immediate postabortion IUD provision into second trimester medical abortion services.

The study objectives were to explore 1) providers and women’s perspectives on preferences, barriers, and facilitators related to postabortion IUD provision, 2) influences on women’s contraceptive decision-making, and 3) the impact of service organization and service context on IUD provision after second trimester medical abortion.

### Study context and RCT procedures

In the study setting, women requesting abortion in the second trimester are referred from their community primary healthcare facilities (CHF) to a centralized hospital for their medical abortion procedure, which involves a regimen of mifepristone followed by misoprostol. In the RCT, women allocated to the immediate arm had an IUD inserted prior to discharge and within 24 h of abortion completion. Those in the delayed arm were given 1 month’s supply of oral contraceptive and referred to their local CHF for IUD insertion 3 weeks later. All women were planned for clinical follow-up at the hospital with an ultrasound examination after 6 weeks. The original RCT included 112 women, 55 and 57 in the immediate (I) and delayed (D) study arms respectively. By intention to treat (ITT) allocation, 82% (I) and 21% (D) of them received the IUD as planned. At 6 weeks 56% (I) and 19% (D) of them were using the original IUD and 76% (I) and 40% (D) were using either the original, a replacement or a newly placed IUD.

Supplemental trial activities to support the safe and effective implementation of the RCT included addressing issues of demand and supply, and participant safety. These were not part of routine care and included: 1) pre-abortion group contraceptive counselling at the hospital for all women seeking abortion, 2) a booked 6-week consultation with the study clinician instead of a standard un-booked outpatient visit, 3) voice calls and text reminders to reschedule missed appointments. In addition, clinic nurses and hospital resident doctors received orientation and training in IUD insertion and WhatsApp-mediated reminders to perform the insertion on the required day.

## Method

### Study design

We conducted this qualitative study alongside the RCT. We used a qualitative research design informed by a thematic analysis approach [9]. We conducted in-depth interviews with key informants including healthcare staff and purposively selected women participating in the RCT. We developed semi-structured interview guides that were adapted to the role of clinical staff in study procedures, the study arm to which women belonged, and the decisions they made regarding the IUD. The interview content was guided by the following main research questions:

1) What are providers and women’s perspectives on preferences, barriers, and facilitators related to postabortion IUD provision? 2) What factors influence women’s contraceptive-decision making? and 3) How does service organization and service context impact IUD provision after second trimester medical abortion?

Interviews with resident doctors were done after they completed their rotation in the facility and clinic staff were interviewed at close-out of the RCT. Women were offered participation after their 3- and 6-month RCT follow-ups. To reduce observer bias interviews were conducted by staff who had not been involved in the RCT, however none were blinded to RCT outcomes as interview scripts differed accordingly.

### Population and sampling

Eligible women for the RCT were 18 years and older, undergoing medical abortion between 17- and 20-weeks GA, and opting and eligible for the copper IUD as postabortion contraception. For this study, we selected potential participants to ensure that both RCT study arms were represented as well as the range of trial outcomes including use of the original IUD as planned, use of a replacement IUD, or non-use of an IUD. To achieve this representation, we expected that we would need approximately 24 interviewees. We offered participation to all resident doctors and nurses at the hospital providing the second trimester medical abortion care, as well as all primary healthcare nurses who had provided the IUD to 2 or more participants in the delayed arm of the RCT (*n* = 14).

### Data collection and ethics

Interview guides were developed in English and translated into isiXhosa by translators experienced in the field of sexual and reproductive health. Back translation was not performed due to limited resources. A female medical doctor performed the telephone interviews with residents. Qualitative research assistants, fluent in English and IsiXhosa, performed interviews in-person with hospital and clinic nurses and women participants. Interviewers were experienced in qualitative research in the field of sexual and reproductive health and rights and women’s rights to access a safe abortion within the South African context and all underwent training specific to this study. We collected data from women until saturation was achieved and no significant new information emerged. All healthcare staff who were offered participation completed their interview. Interviewees gave written informed consent prior to being interviewed. Women were given a ZAR180 (~ 12 USD) food voucher to compensate for their time and transport costs, healthcare staff were not compensated for their time. Interviews lasted between 30 and 40 min, were recorded, translated into English where needed and transcribed. Data were stored in locked files and password protected computer files. Recordings were deleted once they had been checked after data transcription. The study protocol was approved by the University of Cape Town Health Sciences Human Research Ethics Committee (HREC Ref:821/2018), and the Western Cape Provincial Department of Health (WC_201804_006).

Table 1 shows roles in the service for staff and randomization group and primary outcomes for women participants.

**Table 1:** Healthcare staff and trial participants interviewed regarding implications for service delivery of the intrauterine device (IUD) after second trimester abortion. Description according to designation (staff) or trial randomization group and outcome (women)

**Staff**		
**Facility**	**Designation**	**Number of respondents**
Hospital	Study doctor	1
Hospital	Resident doctor	6
Hospital	Senior registered nurse- ward manager	1
Hospital	Enrolled nurse	1
Hospital	Registered nurse	1
Community healthcare facility (CHF)	Doctor in charge of Family Planning	1
CHF	Registered nurse; abortion care provider	2
CHF	Registered nurse; abortion care manager	1
**Women**		
**Trial randomization group**	**Uptake and use of IUD**	**Number of respondents**
Immediate group	Got IUD as planned, IUD in situ at 6 weeks	6
Immediate group	Got 1st IUD at 6 weeks (clinical contraindication for immediate insertion) or got replacement IUD at 6 weeks (1st IUD expelled)	6
Immediate group	IUD expulsed and not replaced at 6 weeks	1
Delayed group	Got IUD at CHF as planned	3
Delayed group	Did not get IUD at CHF, but got 1st IUD at 6 weeks study visit at hospital	6
Delayed group	Not using an IUD	2

### Data analysis

Two researchers (OS and DC) analysed the text data extracted from interviews by thematic analysis. The analysis process involved the following steps: i) Familiarisation with the data through several readings of the interview transcripts, ii) Preliminary coding using categories drawn from interview guides, iii) Searching for patterns or cross-cutting themes across interviews of both providers and women, iv) Reviewing, defining and naming themes, and v) reporting of results [9]. We coded and categorized the data using NVivo 8 qualitative data analysis software. Some categorizations were revised after discussion with ME.

## Results

We identified three main themes in the analysis: 1) Perspectives on timing for IUD insertion after medical abortion, 2) perspectives on and experiences of follow-up, and 3) contextual factors impacting IUD demand and provision, and several subthemes that are presented below.

### Theme 1: perspectives on timing for IUD insertion after medical abortion

Women’s and staff perspectives on the best time to start with the IUD after medical abortion were informed by personal and clinical factors such as convenience, time saved, and contraceptive effectiveness.

#### Preferences for immediate versus delayed insertion of the IUD

Women generally described a preference for immediate insertion. Some from the delayed group felt the 3-week delay would place them at risk of pregnancy, place additional demands on their time, and involve additional costs which meant they might not go for IUD insertion. This was echoed by providers. Preference for immediate insertion was expressed by two participants as follows:

*“well I was so stressed when I found out that I couldn’t put the IUD immediately because I thought what if in this process while I’m waiting for this IUD, what if something happened, coz it’s very hard for us as married woman to use protection, specially to our husbands because they get to ask the questions….” (Immediate group, crossover to delayed, IUD inserted at 6weeks by study clinician)*

*“I felt happy, I didn’t have a problem that I was going to have it inserted because I wanted to have it inserted immediately, because I already heard how it works and just thought that if it works out for immediately, then I should have it inserted immediately and not waste time.”(Immediate group, IUD expulsed and replaced at 6 weeks)*

The increased efficiency of immediate insertion was expressed by a resident doctor as follows:

*“I think in terms of making sure it gets used, immediate insertion is easier for the woman and it cuts out one follow up visit which makes it easier to put in and use and I think to come back 6 weeks later for an insertion and then come back another 6 weeks later for a string check is very labour intensive. Whereas if it is put in at the time, it’s in, she’s more likely to use it and it just avoids second follow up”. (Resident doctor, hospital)*

#### Barriers to immediate insertion of the IUD

Although most healthcare providers considered immediate insertion a better option, they noted that staffing issues, specifically timely availability of providers competent to perform the IUD insertion could be a barrier to an effective service. Other barriers that emerged were the development of abortion complications preventing immediate insertion of the IUD as planned. These included excessive bleeding, prolonged placental retention, or suspected infection.

One senior nursing sister expressed the difficulty regarding availability of resident doctors to insert the IUD immediately following completion of the abortion as follows:

*“only 1 registrar who is working the weekend, she has an emergency, she has theatre that she must take up, she has 2 wards that she must look after. And then apparently there’s this patient that is waiting to go home, panicking because (the IUD insertion) it’s not done, …….so if they delaying and all of that and she’s done, she would rather take an injection or something else because the patient wants to go home”. (Sister in charge of ward, hospital)*

#### Mechanisms to facilitate immediate insertion of the IUD

##### Training of providers

The need for competence and confidence in IUD insertion was a recurring theme expressed by providers, and emerged directly from the supplementary trial activities, notably the IUD insertion training sessions. These had a positive impact on motivation to provide immediate IUD insertion as expressed by one provider:

“*well, I think if we had a session like that, we’d definitely be more confident in putting in the IUD and ja, I think it would improve the number of patients getting IUDs on time. I did do the training session that we had, and I’d been putting them in before that, but it was a nice refresher, I felt more confident after that*” *(Resident doctor, hospital)*

##### Reminders to doctors to insert the IUD immediately postabortion

Providers considered a WhatsApp group a good mechanism to remind each other about patients needing the IUD inserted, especially on weekends, but also feared it may not be sustainable:

*“I mean she’s getting a TOP and she needs an IUD. So, she* [the study clinician] *would usually remind you the day before and especially on the weekends, I think it was really, really helpful because you wouldn’t know that whether there’s a TOP in the, in the ward or not coz they’re totally closed off from everyone else” (Resident doctor, hospital)*

##### Task-shifting provision of the IUD to other cadres of providers

To resolve staff shortages and lack of training, some healthcare staff suggested having a dedicated trained midwife to provide abortion and postabortion care or alternatively training interns to do the IUD insertions as this should be within their competency and they would be more available.

*“I think it would be solved by having a, a qualified nurse or qualified midwife, just one person who’s responsible for this stream of care and then that would also mean that these patients can go home on the same day” (Study clinician)*

*“I think it would have been helpful if the interns was maybe trained to have done it because there’s more interns than registrars”. (Ward operations manager, hospital)*

### Theme 2 perspectives on and experiences of follow-up

#### Continuity of care following abortion

In the study’s immediate arm protocol the abortion and postabortion care are rendered at the same place and by a provider familiar with the woman’s reproductive history which meant she need not explain herself to a new healthcare provider:

*“I mean, a great benefit is that women actually get the IUD inserted. And I think there’s a second advantage because once women have it and are sort of in the system, they are also more likely to come back to their follow up, at least if you provide continuity of care. If you can offer that, women stay in the fold, you have a relationship and you say come back see me, I know your story, I know you’ve had an abortion, there’s no problem with that, then I don’t think they’re as reluctant to come back for a check-up”. (Study clinician)*

#### Women’s decisions regarding the IUD during study follow-up

Of the women in the RCT immediate group who experienced partial expulsion of the IUD, most requested a replacement IUD at 6 weeks. Some women in the delayed group asked for an IUD at the 6-week follow-up even if they did not go to the CHF for insertion as planned. Among the women in the delayed arm who did not have the IUD inserted as planned, many reverted to short term methods they had used before.

One woman who requested an IUD replacement at 6 weeks because the original IUD was malpositioned said the following:

*“of course, I did have some problems after I put the IUD……she said to me, it’s low. Do you want us to take it right now, we put it right now or you want us to make another appointment). Then I said no, if you’ve got a chance you can do it now because I was already on a bed. And then it was not sore anywhere honestly, and then I decided immediately, take it out and put the new one.* **(***Immediate group, IUD malpositioned, replaced at 6 weeks***)**

Another woman was planned for insertion at 3 weeks, but the provider was not available at the clinic. At her 6-week follow-up with the study clinician she requested an IUD; she described what happened as follows:

*“I went to the clinic but the lady that was implanting the IUD wasn’t there, so that’s why I didn’t put it in, they gave me the injection for 3 months and they said I must come back January…. But I call the lady from [hospital name] and said, and she said it’s fine, I can come, they will do it for me there…... (Delayed group; IUD inserted at 6 weeks by study clinician)*

A woman in the delayed group changed her mind about the IUD but completed her follow-up interviews by phone. She explained her decision for the injectable method as being concerned about the possibility of expelling the device, not noticing it, and becoming pregnant again.

*“Yes, that while I have it in, it might fall out and what if I’m not as lucky as the others where when it falls out, they see it in their panties, and I don’t see it and then I continue to have sex and then fall pregnant again, thinking that it’s still in there whereas it had fallen out a long time ago.” (Delayed group; injectable on discharge, no IUD at 6weeks, 3 months or 6 months)*

Many women who had the IUD inserted subsequently expressed happiness with the method and feelings of autonomy and liberation.

*‘this IUD, it’s quite good to me. I do not have even any cramps; my menstruation periods are normal. I do not have even any problem with it, it is fine, to me it’s fine. I just like it, I’m not using anything, I can’t even feel it, I can’t even feel anything except when I put a finger inside for me to, to check. Yes, that’s when I feel it you know inside. But it’s as normal fine” (Immediate, got IUD as planned, IUD in situ throughout)*

*“ooh I felt really, I’m, I’m so happy, I feel free and stress free. I didn’t, I don’t have anything to worry about. About like kids, uh huh, the ones I have now it’s fine, so I’m free, safe and free (Delayed group, IUD placed at 6 weeks by study clinician)*

### Theme 3: contextual factors impacting IUD demand and provision

#### Pre abortion counselling

Women reported that the group counselling session at the abortion facility supported contraception decision-making by providing information, encouragement, and reassurance which had a positive effect on IUD uptake and continuation. For instance, a participant noted:

*“I took her word for it and I tried it and it’s still working for me. She convinced me that it is a better option because she understands how I feel when I told her about the other ones I was on” (Immediate group, IUD expulsed and replaced at 6 weeks)*

#### Resource constraints and staff shortages

Lack of availability of resources was a frustration expressed by healthcare staff and a potential barrier to providing the IUD immediately after the abortion, as expressed by two different doctors:

“*it’s uncomplicated, it’s fairly easy. The only difficulty is just getting the necessary equipment*…. *And then at the beginning of the study, we also had a struggle with the instruments until they make a plan of getting their own instruments”. (Resident doctor, hospital)*

*“I think there might be opposition from people because they see it as an extra job or chore to do, just because it takes more time than writing up a script for Petogen or the pill. But I don’t think that should be a reason why it’s not implemented” (Resident doctor, hospital)*

#### Women’s negative perceptions of primary healthcare services

Women explained reluctance to attend CHFs for follow-up because they feared facing a judgmental attitude on the part of healthcare providers. They also noted that waiting times at clinics were long and some described being turned away more than once.

*“Like for example, going to the local clinics, sisters will ask you, ask you why, what happened, why you making abortion, you know this thing – like they will make you feel bad about doing it and, which is unprofessional, they not supposed to do it.*” *(Delayed group, IUD placed at 6 weeks by study clinician)*

## Discussion

### Main findings

We found that immediate insertion of the IUD is generally preferred by both women and clinical staff for reasons of convenience and continuity of care, and results in higher rates of clinical follow-up. Most women who receive the IUD are highly satisfied. The need for continuous training, communication mechanisms, instruments, and available staff in a highly pressured health system, as well as the rote administration of contraception, may be barriers to implementation.

### Interpretation

We found that if women receive an IUD after the abortion they are motivated to continue even if they require a replacement, and express high satisfaction with this method. Women expressed a clear preference for immediate over delayed IUD insertion and both women and staff predicted that delayed insertion was unlikely to occur because of the inconvenience, cost, and potential negative experience of following up at referral clinics.

The study-initiated communication system with doctors was an important factor to ensure the IUD was inserted on time. Nurses highlighted the fact that doctors were absent from the ward during the time when most women would optimally receive their IUD for them to be able to go home the same day. Nurses and doctors also noted that staff shortages and lack of training were barriers to immediate IUD insertion. Making immediate IUD insertion part of routine postabortion care in the second trimester may require a restructuring of services to task-shift IUD insertion to either trained nurses or interns with higher presence in the ward, as well as regular training of clinic staff to maintain confidence in recommending and providing the IUD. Lack of recurring training is a known barrier to sustainable reproductive health services including IUD insertion [10, 11]. Trained nurses and midwives can however provide equally safe, efficient, and acceptable abortion and contraception care to physicians and their role may be better suited to person-centred services [12–14].

With the higher risk of IUD expulsion after immediate insertion, it is of additional importance that women attend their clinical follow-up. Women expressed reluctance to go to their referral clinics because they feared judgement on the part of providers and were therefore unwilling to tell their abortion history again. In contrast, the RCT follow-up structure with study staff was adhered to. The continuity of care and booked follow-up appointments offered during the study were perceived by women as personalized and convenient follow-up with non-judgmental communication, and are therefore important to consider when implementing immediate insertion into routine service delivery. A review of current evidence supports that person-centred care is central to, and often lacking, in abortion care and impacts on quality of care [15].

The group contraceptive counselling increased interest and uptake of the IUD. Providers are often unfamiliar with this method, and women do not have family members or friends who can vouch for it [5, 7] which may contribute to its low use in South Africa. Our findings are in line with a previous study that has shown that the more knowledgeable a health care provider is about the IUD, the more likely they are to recommend it [16]. In the original RCT we found that administration of the progestin injectable was such a routine part of postabortion care that it was recurrently administered, in deviation of protocol, despite multiple reminders. Familiarization and training on IUD insertion is likely a core component of increasing demand and immediate initiation of the IUD postabortion.

Access to care is a multifaceted process which requires an interplay between laws and policies, provider attitudes, service delivery, and patient behaviours, in the absence of which access is jeopardized [17]. Implementation of immediate IUD insertion postabortion must be informed by an understanding of service delivery barriers, factors affecting women’s health seeking behaviour, and how these may influence service delivery.

### Strengths and limitations

This qualitative study was designed and performed concurrently with the clinical trial, the interviews as well as the main data analysis were performed by researchers not involved in trial procedures, and participants represented a broad range of roles and experiences. All doctors and most nurses involved in the clinical care relevant to the RCT were interviewed. Bias in interpretation was mitigated by researcher triangulation but cannot be excluded. The identified barriers and facilitators to immediate IUD insertion are contextual to South Africa but many are likely applicable to similar settings.

## Conclusions

Both women and providers support immediate insertion of the IUD after second trimester abortion. Adequate staffing and/or task-shifting of IUD insertion, available instruments and continuous training are important pre-requisites for implementation in the opinion of providers. For women, continuity of care, non-judgemental communication and convenient follow-up are important factors in ensuring uptake and adherence to follow-up.

## Data Availability

All data will be made available by the corresponding author upon reasonable request.

## Abbreviations

LARC: Long-acting reversible contraceptives/contraception
RCT: Randomized controlled trial
IUD: Intrauterine device
MA: Medical abortion
GA: Gestational age
CHF: Community health facilities
PE: Process evaluation
ZAR: South African Rands (currency)
USD: United States dollars (currency)
ITT: Intention to treat
I: Immediate
D: Delayed
OCs: Oral contraceptives
